# Causes of COVID-19 Outbreaks During Sports and Exercise: A Systematic Review

**DOI:** 10.1007/s40279-024-02153-7

**Published:** 2024-12-11

**Authors:** Masaki Machida, Koichi Dai, Itaru Nakamura, Shigeru Inoue

**Affiliations:** 1 Department of Preventive Medicine and Public Health, Tokyo Medical University, 6-1-1 Shinjuku, Shinjuku-ku, Tokyo, 160-8402 Japan; 2 Department of Infection Prevention and Control, Tokyo Medical University Hospital, 6-7-1 Nishishinjuku, Shinjuku-ku, Tokyo, 160-0023 Japan

## Abstract

**Background:**

Physical activity is beneficial for preventing non-communicable and infectious diseases, such as pneumonia. Physical activity is also a potential protective factor for reducing coronavirus disease 2019 (COVID-19) severity. Conversely, outbreaks of respiratory viral infections are more likely to occur owing to group activities, opportunities for contact with individuals and vocalisations. Since the onset of the COVID-19 pandemic, several cases of COVID-19 outbreaks during various sports and exercise have been reported. However, the common causes underlying these outbreaks remain unclear.

**Objective:**

The objective of this study is to identify the causes of COVID-19 outbreaks during sports and exercise using systematic review approach.

**Methods:**

Our eligibility criteria were published articles reporting case investigation on COVID-19 outbreaks and the cause during sports and exercise. Studies such as reviews and observational studies without case investigations were excluded. PubMed, CINAHL, WHO COVID-19 Research Database and Ichushi Web were searched on 28 August 2023. The quality of included studies was rated using a quality criteria checklist adapted from a previous systematic review of influenza outbreaks. Vote counting of outbreak causes was performed by type of sports (team or individual).

**Results:**

Twenty-one articles reporting 22 outbreaks were identified (quality: high, 9; medium, 9; and low, 3). The outbreaks were most frequently reported in fitness classes, followed by soccer. Most studies listed multiple causes of the outbreaks. The most common suspected cause of outbreaks in individual exercise, mostly from fitness classes, was poor ventilation and not wearing masks, followed by not maintaining physical distance and participation of individuals with some symptoms. In team sports, the most common cause was interaction outside the game, such as social events.

**Conclusions:**

This systematic review found a limited number of case investigations suggesting that COVID-19 outbreaks during sports and exercise may be associated with the inhalation of aerosols in indoor settings, interactions outside of team sports games and participation of individuals with some symptoms. Prevention strategies that focus on mitigating these issues may be effective at preventing sports and exercise-associated respiratory infectious diseases outbreaks.

**PROSPERO Registration Number:**

CRD42023443158.

**Supplementary Information:**

The online version contains supplementary material available at 10.1007/s40279-024-02153-7.

## Key Points

**Table:** 

The results showed that the inhalation of aerosols in indoor settings, interactions outside of exercise (e.g. social events and eating together) and participation of individuals with symptoms were common causes of outbreaks in sports and exercise.
Contact transmission during sports and exercise may not be an important factor in COVID-19 outbreaks.

## Introduction

Physical activity is beneficial for preventing non-communicable and infectious diseases, such as pneumonia [1–3]. Regarding respiratory viral infections that cause pandemics, physical activity is also a potential factor in reducing coronavirus disease 2019 (COVID-19) severity [4–8]. However, opportunities for physical activity were reduced during a pandemic because of the recommendation to reduce personal contact to prevent person-to-person transmission [9–11]. Specifically, sports were often required to be refrained from as they are prone to infectious disease outbreaks [12, 13]. In sports activities, outbreaks of respiratory viral infections are more likely to occur owing to group activities, opportunities for contact with individuals and vocalisations [14, 15]. During the COVID-19 pandemic, several cases of COVID-19 outbreaks during various sports and exercise were [16]. In professional sports, there are several instances in which strict and multicomponent infection prevention strategies, such as the bubble protocol and periodic screening tests, have allowed sports events to be conducted without outbreaks during the COVID-19 pandemic [17, 18]. However, these strategies involve enormous costs and manpower, making them difficult to implement in amateur and recreational settings. Feasible and practical prevention strategies are required for citizens to continue exercising and performing sports during an outbreak or pandemic of a respiratory virus infection, such as COVID-19. Several studies have reported causes of outbreaks during sports and exercise [19–39]; however, common causes remain unclear. Identifying the common causes of outbreaks is important for key non-pharmaceutical interventions (NPIs) to mitigate outbreaks.

In this review, we aim to systematically identify COVID-19 outbreak cases during sports and exercise and to clarify the causes of these outbreaks.

## Methods

We used a systematic review approach following the synthesis without meta-analysis (SWiM) guideline [40] and reported the results according to the Preferred Reporting Items for Systematic Reviews and Meta-Analyses (PRISMA) 2020 guidelines [41]. The protocol for this review was registered in PROSPERO before screening took place (registration number: CRD42023443158) [42].

### Eligibility Criteria and Search Strategy

Our eligibility criteria for the study selection were published articles reporting on COVID-19 outbreaks and their causes during sports and exercise. In this review, a COVID-19 outbreak was defined as two or more confirmed cases of COVID-19 in a facility or group with onset within a specific time period (approximately 14 days). In this review, a case investigation was also defined to detect and notify individuals with suspected or confirmed infections and to attempt to identify the cause of the outbreak [43, 44]. We included only studies that reported case investigations in English or Japanese. We excluded systematic and narrative reviews, guidelines, opinion pieces, conference proceedings, preprint articles, intervention studies, observational studies without case investigations, laboratory or virological studies and animal studies. We conducted electronic searches in PubMed, Cumulative Index to Nursing and Allied Health Literature (CINAHL, via EBSCOhost), WHO COVID-19 Research Database and Ichushi Web. Ichushi Web is a Japanese bibliographic database updated by the Japan Medical Abstracts Society. The search was conducted on 28 August 2023. The search strategy was developed by one infectious disease and physical activity researcher (M.M.) and one information specialist (librarian). We validated our search terms in PubMed by testing their ability to retrieve four specific studies known to be relevant to our research focus [20–22, 38]. These studies served as benchmarks to ensure the effectiveness of our search strategy in identifying key literature related on COVID-19 outbreaks cases during sports and exercise. Candidate search terms were identified by examining words in the titles, abstracts and subject indexes of the records. A draft search term was developed using these terms, and additional search terms were derived from the results of this strategy. The final search terms are listed in Supplementary Table 1. The search terms were validated by testing whether they could identify four known relevant studies and five studies that were identified as part of the strategy development process [19–22, 25, 34, 36–38]. All nine studies were identified by the search strategies in PubMed and the WHO COVID-19 Research Database. The final search strategy was reviewed by the researcher and librarian using the Peer Review of Electronic Search Strategies (PRESS) 2015 guideline evidence-based checklist [45].

### Selection and Data Collection Process

After removing duplicate records, two researchers (M.M. and K.D.) independently reviewed the titles and abstracts of the first 100 records and discussed any inconsistencies until a consensus was reached. Subsequently, the researchers independently screened the titles and abstracts of all retrieved articles. In cases of disagreement, a consensus on which articles to screen for full text was reached by discussion. Two researchers (M.M. and K.D.) independently screened full texts for inclusion. In cases of disagreement, a consensus was reached on inclusion or exclusion by discussion. Screening was performed using the Rayyan Systems online screening tool [46].

Two researchers (M.M. and K.D.) independently extracted the following data from each paper into a prepared data extraction form in Excel spreadsheets: publication details (author and location), sports and exercise characteristics (e.g. type, setting and outbreak date); infection prevention strategy at the outbreak setting (wearing a mask, physical distance, ventilation, hand hygiene, surface disinfection, bubble protocol, limitations on capacity, temperature check, physical condition self-check, periodic laboratory screening and vaccination); details of case investigation (e.g. case investigation method, timeliness of case investigation, proportion of participants followed up, confirmation methods of COVID-19, number of COVID-19 positives and attack rate [the number of COVID-19 positives divided by total participants]), outbreak causes and rationale for the cause. The extracted data were compared, and any discrepancies were resolved through discussion.

### Quality Assessment and Data Synthesis

We assessed the quality of each included study using an original quality criteria checklist adapted from a checklist used in a previous systematic review of aircraft influenza transmission [47]. A detailed checklist is provided in Supplementary Table 2. Two review authors independently applied the tool to each included study.

In the data synthesis, we did not perform a meta-analysis because of the characteristics of case investigation studies, such as a large number of incompletely reported outcomes or effect estimates and clinical and methodological diversity [48]. We synthesised the extracted data based on the SWiM guideline [40]. Vote counting was used to synthesise the cause of the outbreak and classify the study into team sports and individual sports or exercise [48]. The results were visualised using harvest plots [49, 50].

### Equity, Diversity and Inclusion Statement

The author group comprised junior, mid-career and senior researchers from different disciplines with diverse cultural backgrounds. Inclusive data collection methods were used to capture evidence from various populations irrespective of sex, age, race, type of sport, competition level, location, education or socioeconomic status.

## Results

### Study Selection

**Fig. 1:**
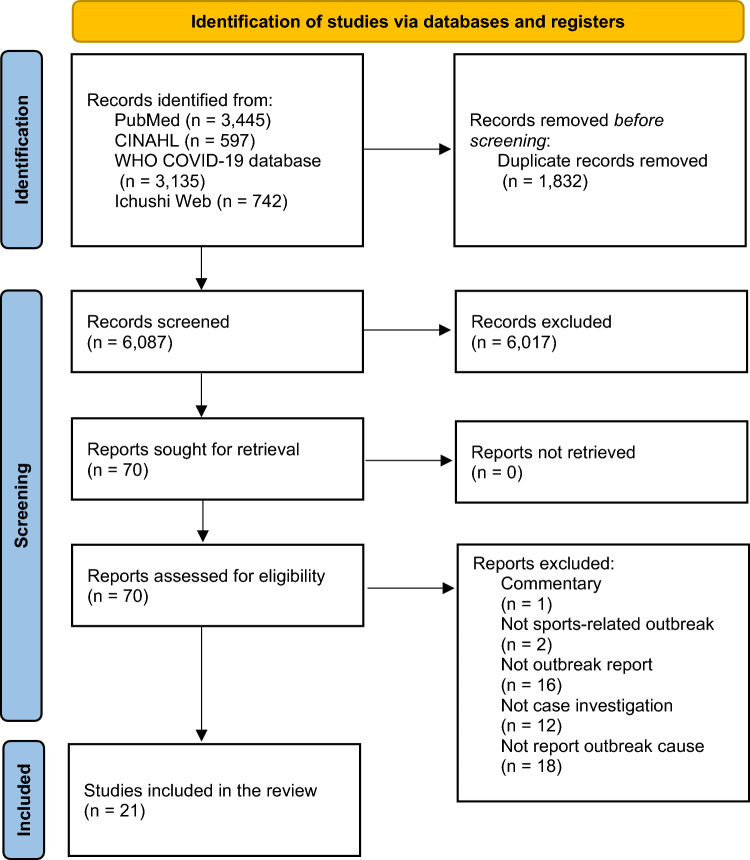
Identification and selection of studies for the review

We found 7919 records in the database search. After removing duplicates, we screened 6087 records, from which we reviewed 70 full-text documents and finally included 22 outbreak reports related to 21 studies (Fig. 1) [19–39]. In the study by Schumacher et al., two distinct outbreak cases were reported within the same research context [30]. We treated these as separate data because each outbreak had unique causes that were extracted and evaluated independently.

Of the 70 full-text documents, 10 were excluded for the following reasons: unclear whether an outbreak occurred (*n* = 3) [51–53], unclear whether a case investigation was conducted (*n* = 1) [54] and unclear cause of the outbreak (*n* = 6) [55–60]. Carson et al. reported an outbreak at a dance camp event [61]. However, we excluded this study from our review because it involved only an anonymous self-reported questionnaire survey.

### Study Characteristics

Table 1 displays for each included COVID-19 outbreak case the citation, outbreak location and setting, number of participants, number of cases, outbreak cause and rationale for it. Ten outbreaks were reported in America: two in Canada, China, Germany, South Korea and Qatar and one in Slovenia and the United Kingdom. The number of reported outbreaks was highest in the fitness class (five cases) [19–23], followed by soccer (four cases) [29–31] and American Football [32, 33] and ice hockey [34, 35] (two cases). Other outbreaks were reported in various sports, such as baseball, basketball, curling, jogging, taekwondo and volleyball. Several studies reported outbreak cases in professional athletes during the season [30, 31, 33, 37, 39]. Most of the reported outbreaks occurred in 2020. In most outbreaks, participants did not wear masks during exercise (Supplementary Table 3).

**Table 1:** Summary of included COVID-19 outbreak cases ordered based on type of sports and exercise

	Study (quality rating)	Outbreak location	Type	Setting	Date	Number of participants	Number of cases (attack rate)	Outbreak cause	Rationale for the cause
Individual sports or exercise									
	Jang et al. [19] (low)	South Korea	Fitness class	Fitness facility	2020.2–3	217	57 (26.3)	Exercise in crowded places, high-intensity exercise	Exercise in crowded places: no one is infected in a small class High-intensity exercise: there was no case in pilates and yoga classes
	Groves et al. [20] (high)	USA	Fitness class	Fitness facility	2020.6–7	62	21 (33.9)	Not wearing masks during exercise, poor ventilation, not maintaining physical distance during exercise, exercise with shouting	NP
	Lendacki et al. [21] (medium)	USA	Fitness class	Fitness facility	2020.8–9	81	49 (60.5)	Not wearing masks during exercise, poor ventilation, participation of individuals with some symptoms	Not wearing masks during exercise: the odds ratio was high, although not significant
	Bart et al. [22] (low)	USA	Fitness class	Fitness facility	2020	7	3 (42.9)	Not wearing masks during exercise	NP
	Chu et al. [23] (high)	China	Fitness class	Fitness facility	2021.2–3	301	102 (33.9)	Not wearing masks during exercise, poor ventilation	NP
	Anderson et al. [24] (high)	Canada	Workout	Fitness facility	2020.9–10	251	27 (10.8)	Poor ventilation	NP
	Brlek et al. [25] (medium)	Slovenia	Squash	Squash court	2020.3	6	5 (83.3)	Poor ventilation, poor surface disinfection, high-intensity exercise	NP
	Shin et al. [26] (medium)	South Korea	Taekwondo class	Taekwondo gym	2021.1–2	108	30 (27.8)	Food consumption inside the gym and teatime, participation of individuals with some symptoms	Food consumption inside the gym: odds ratio was significantly higher
	Dougherty et al. [27] (medium)	USA	Gymnastics class	Gymnastics facility	2021.4–5	133	26 (19.5)	Not wearing masks during exercise, poor ventilation, poor surface disinfection, participation of individuals with some symptoms, Low vaccination	NP
	Qi et al. [28] (high)	China	Jogging	Outdoor park	2022.8	2836	39 (1.4)	Not wearing masks during exercise, not maintaining physical distance during exercise	Not wearing masks during exercise: most of the patients were not wearing masks Not maintaining physical distance during exercise: there were several positives among those who did not maintain physical distance
Team Sports									
	Teran et al. [29] (high)	USA	Soccer	Voluntary training session in university team	2020.7–8	45	17 (37.8)	Birthday party, living together in a shared house	Birthday party: all seven birthday party attendees were positive Living together in a shared house: odds ratio was significantly higher
	Schumacher et al. [30] (medium)	Qatar	Soccer	Professional sports season	2020	NP	2 (NP)	Sharing a car	No other teammates were infected
	Schumacher et al. [30] (medium)	Qatar	Soccer	Professional sports season	2020	NP	4 (NP)	Private dinner	None of the other teammates were subsequently found to be infected
	Basu et al. [31] (medium)	United Kingdom	Soccer	Professional sports season	2020	NP	4 (NP)	Contact with an infected individual in the community	There were no infected individuals who had no contact outside the game
	Siegel et al. [32] (medium)	USA	American Football	High school environment	2020.9–10	54	14 (25.9)	Not wearing masks during exercise, poor surface disinfection, bus transportation	NP
	Mack et al. [33] (high)	USA	American Football	Professional sports season	2020.9–10	NP	21 (NP)	Not wearing masks outside of exercise, not maintaining physical distance outside of exercise, eating in a facility	NP
	Atrubin et al. [34] (high)	USA	Ice hockey	Ice rink	2020.6	22	14 (63.6)	Not maintaining physical distance during exercise, not maintaining physical distance outside of exercise	NP
	Krug et al. [35] (low)	USA	Ice hockey	Ice rink	2020.10–11	NP	33 (NP)	Indoor picture night	The peak of the outbreak was confirmed 5 days after the picture night
	Burak et al. [36] (medium)	Canada	Curling	Curling rink	2020.3	73	56 (76.7)	Social events (e.g. buffet lunches at carling rink), participation of individuals with some symptoms	Buffet lunches at carling rink: significantly more of the positive participants attended the lunch buffet There were no cases of the team who did not participate in all social events
	Morath et al. [37] (high)	Germany	Volleyball	Professional sports season	2020.9	15	5 (33.3)	Social gathering in a bar	None of the individuals who did not attend the bar event tested positive
	Pauser et al. [38] (medium)	Germany	Basketball	Indoor basket court	2020.11	69	36 (52.2)	Not wearing masks during exercise, not wearing masks outside of exercise	The number of positive cases was high among those who did not wear masks
	Murray et al. [39] (high)	USA	Baseball	Professional sports season	2020	146	21 (14.4)	Interactions outside of on-field play	None of the players on the opposing team were infected

*NP* not provided

### Quality Assessment

Nine studies were rated as high quality [20, 23, 24, 28, 29, 33, 34, 37, 39], nine as medium quality [21, 25–27, 30–32, 36, 38] and three as low quality [19, 22, 35]. Supplementary Table 4 provides details of the quality ratings.

### Causes of Outbreak

**Fig. 2:**
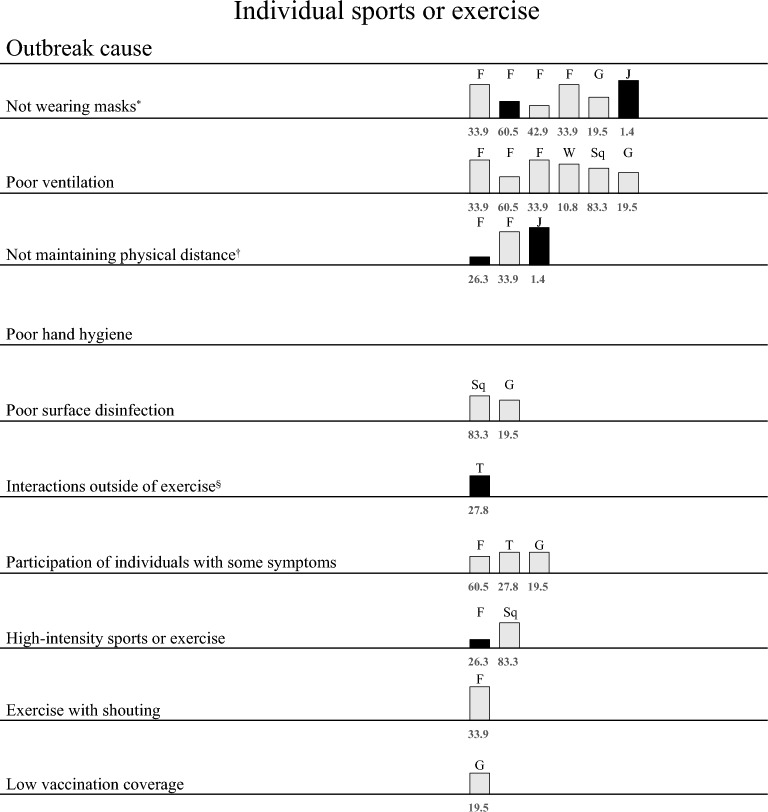
Coronavirus disease 2019 (COVID-19) outbreak causes in individual sports or exercise. Ten rows covering all categories of COVID-19 outbreak causes were described in the reviewed studies. All the causes listed in the reviewed studies are placed in rows. The rating score for the study quality is indicated by the height of the bar. Each bar is annotated according to the type of sport involved. Causes for describing the rationale in the study are indicated with full-tone (black) bars and causes without describing the rationale with half-tone (grey) bars. The number below the bar indicates the attack rate. *NP* not provided, *F* fitness class, *G* gymnastics class, *J* jogging, *Sq* squash, *T* taekwondo class, *W* workout. *Included not wearing masks during exercise and not wearing masks outside of exercise. ^†^Included not maintaining physical distance during exercise, not maintaining physical distance outside of exercise and exercising in crowded places. ^§^Included food consumption inside the gym and teatime

**Fig. 3:**
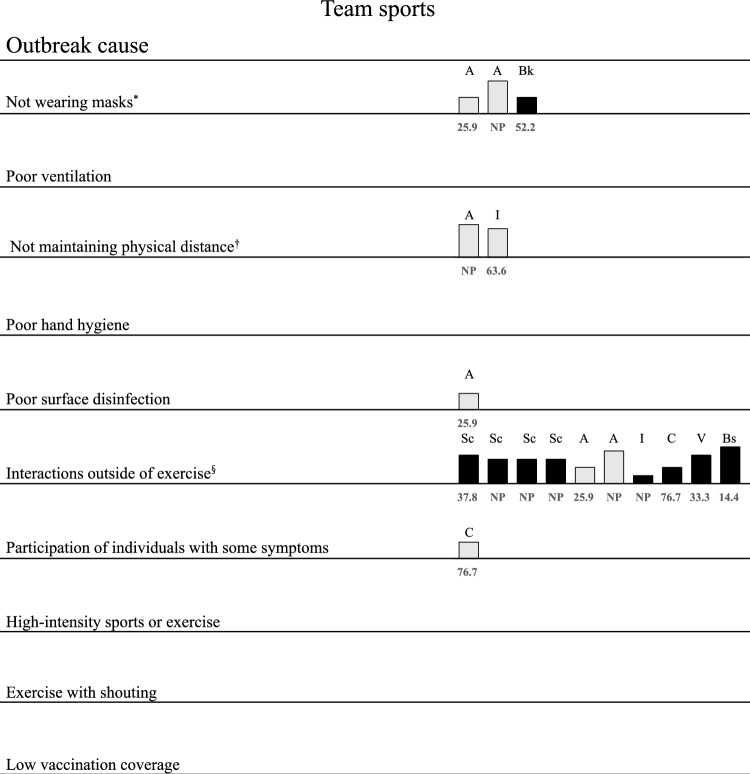
Coronavirus disease 2019 (COVID-19) outbreak causes in team sports. Ten rows covering all categories of COVID-19 outbreak causes were described in the reviewed studies. All the causes listed in the reviewed studies are placed in rows. The rating score for the study quality is indicated by the height of the bar. Each bar is annotated according to the type of sport involved. Causes for describing the rationale in the study are indicated with full-tone (black) bars and causes without describing the rationale with half-tone (grey) bars. The number below the bar indicates the attack rate. *NP* not provided, *A* American Football, *Bk* basketball, *Bs* baseball, *C* curling, *I* ice hockey, *Sc* soccer, *V* volleyball. *Included not wearing masks during exercise and not wearing masks outside of exercise. ^†^Included not maintaining physical distance during exercise and not maintaining physical distance outside of exercise. ^§^Included birthday parties, living together in a shared house, sharing a car, private dinner, contact with an infected individual in the community, bus transportation, eating in a facility, indoor picture night, social events, social gatherings in a bar and interactions outside of on-field play

Table 1 provides a summary of the included studies, ordered by the type of sports and exercise. Most outbreak reports listed multiple causes. Several studies had insufficient or missing information regarding the rationale for the cause of the outbreak [20, 22–25, 27, 32–34]. Figures 2 and 3 show a harvest plot of the results of vote counting by categorising these causes.

#### Not Wearing Masks

Six individual exercise [20–23, 27, 28] and three team sports [32, 33, 38] case investigations reported not wearing masks as the cause of the outbreak. Five of the six outbreaks occurred in indoor fitness or gymnastics facilities [20–23, 27], and several studies reported poor ventilation as a cause of the outbreaks, along with not wearing masks [20, 21, 23, 27]. Qi et al. reported an outbreak in an outdoor park with an individual who was jogging as the primary case [28]. This study reported that although the attack rate of the outbreak was low (1.4%), several infected individuals did not maintain physical distance and most were not wearing masks [28]. However, the proportion of infected individuals who were wearing masks was not clearly documented. In team sports, two case investigations of American Football reported that not wearing a mask was the cause; however, no rationale was stated [32, 33]. Outbreaks at professional basketball games had more infected cases among non-mask wearers than mask wearers (no statistical analysis) [38].

#### Poor Ventilation

Six individual exercise [20, 21, 23–25, 27] case investigations reported poor ventilation as the cause of the outbreaks. Five of the six outbreaks occurred in fitness or gymnastics facilities [20, 21, 23, 24, 27]. Brlek et al. reported an outbreak in squash during the early phase of the COVID-19 pandemic [25]. This case investigation reported transmission to the primary opponent and players who used the same court after the primary case. No rationale was provided for any of the case investigations.

#### Not Maintaining Physical Distance

Three individual exercise [19, 20, 28] and two team sports [33, 34] case investigations reported that not maintaining physical distance was the cause of outbreaks. Jang et al. reported a large-scale outbreak in South Korea during the early phase of the COVID-19 pandemic [19]. The report indicated that there were no infected individuals in small fitness classes, suggesting the importance of maintaining physical distance. In team sports, not maintaining physical distance was pointed out as a cause in case investigations of American Football and ice hockey; however, the rationale for this was not described [33, 34].

#### Poor Hand Hygiene and Poor Surface Disinfection

No case investigation reported poor hand hygiene as the cause of the outbreaks. Three case investigations reported poor surface disinfection as the cause of the outbreak; however, no rationale for this was described [25, 27, 32]. Brlek et al., who reported an outbreak in squash, speculated on the possibility of poor ventilation and poor surface disinfection as the causes of the outbreak [25]. Dougherty et al., who reported an outbreak at a gymnastics facility, also speculated on multiple causes of the outbreak in addition to poor surface disinfection as the cause of the outbreak [27].

#### Interactions Outside of Exercise

One individual exercise [26] and ten team sports [29–33, 35–37, 39] case investigations reported interactions outside of exercise as the cause of the outbreaks. Shin et al. reported that in an outbreak in a taekwondo class, there were significantly more COVID-19 cases among those who had eaten in the facility and that all members of the class who had eaten at teatime were infected [26]. Outbreak case investigations during the professional sports season in volleyball and soccer reported that COVID-19 cases occurred only among those who had interactions outside the game, such as dinner parties and car sharing [30, 31, 37]. In an outbreak at a recreational curling event, Burak et al. reported significantly more COVID-19 cases among those who attended a lunch buffet and no cases in a team that did not attend all social events [36]. The outbreak case reports in baseball, soccer and American Football included some matches during the outbreak; however, no transmission of COVID-19 to the opposing team occurred [30, 32, 39].

#### Participation of Individuals with Some Symptoms

Three individual exercise [21, 26, 27] and one team sports [36] case investigation reported participation of individuals with some symptoms as the cause of the outbreaks. One case in a fitness facility reported by Lendacki et al. showed that two attendees participated in multiple fitness classes while being symptomatic, although they were screened for symptoms at entry [21]. Burak et al. reported that 10 (13.7%) participants in the curling bonspiel where the outbreak occurred participated in curling with mild symptoms [36]. In addition, one fitness facility outbreak [19] and an outdoor park outbreak [28] reported individuals with mildly symptomatic COVID-19 participating in exercise.

#### High-Intensity Exercise and Exercise with Shouting

Two individual exercise [19, 25] case investigations reported high-intensity exercise as the cause of the outbreak. The fitness facility where Jang et al. reported a major outbreak held several fitness classes [19]. The outbreak occurred mainly among the participants and instructors in the fitness dance class; however, there were no cases in the Pilates and yoga classes. Thus, Jang et al. hypothesised that lower-intensity Pilates and yoga did not cause the same transmission effects as the more intense fitness dance class [19].

One case investigation in a fitness facility reported exercise with shouting as the cause of the outbreak [20]. This case investigation reported an outbreak in a 1 h stationary cycling class in which the instructor, who had COVID-19, shouted and the attack rate was 100% [20].

#### Low Vaccination Coverage

Most studies included in the review reported outbreaks in 2020 before the COVID-19 vaccine became widely available. Only one case investigation in a fitness facility reported low vaccination coverage as a cause of the outbreaks. Dougherty et al. reported that 85% of patients with COVID-19 cases were unvaccinated in the outbreak (vaccination coverage among those who were not patients was not known) [27].

## Discussion

This systematic review of case investigations identified several factors associated with COVID-19 transmission during sports and exercise, including participation of individuals with some symptoms and interactions outside of exercise, such as dining and car sharing. Other factors may increase the likelihood of transmission, such as small, confined spaces with poor ventilation, not wearing masks and intense vocalization (such as shouting) in indoor settings.

Several case investigations, especially in fitness facilities, reported not wearing masks, poor ventilation and not maintaining physical distance as the main causes of the outbreak [19–24, 27]. Recently, the traditional concept of transmission routes of respiratory infection (i.e. droplet, airborne and contact transmission) has been noted to be inadequate, and a new definition has been proposed (i.e. inhalation, spray and touch) [62]. As of 2023, one of the main routes of transmission of respiratory infectious diseases, including COVID-19, is thought to be inhalation of aerosol [62, 63]. Wearing a mask and ventilation are typical measures to reduce transmission by inhalation and maintaining physical distance is designed to reduce transmission by spray of large droplets [62]. However, this study showed that they were not adequately implemented in several outbreak settings, especially in indoor fitness facilities. NPIs that focus on reducing transmission by inhalation can mitigate outbreaks, even if cases of infection have been discovered during exercise and sports [64, 65]. Correct mask wearing is difficult in many daily life situations [66] and even more difficult during exercise and sports. Additionally, evidence on the effectiveness of masks is lacking [67]. Conversely, ventilation is gaining increased attention for its role in preventing respiratory infections [63], offering a low-cost NPI for individuals while addressing long-distance airborne transmission of COVID-19, both during and outside of sports in indoor settings [68]. Thus, NPIs that focus on reducing transmission by inhalation, especially ventilation, may be particularly important for preventing infection during sports and exercise.

One case investigation at a fitness facility in South Korea reported differences in attack rates according to the intensity level of physical activity [19]. This case investigation reported lower attack rates in yoga and Pilates classes than in dance fitness classes. Metabolic equivalents (METs) are approximately 2.5–4.0 METs for yoga and Pilates and approximately 5.0–8.0 METs for dance [69]. A previous simulation study estimated that the viral load emitted by infected individuals increased with the intensity of physical activity [70]. Therefore, in addition to improved ventilation, relocating vigorous physical activities outdoors or limiting the intensity of indoor physical activity may be effective in preventing outbreaks during sports and exercise. Furthermore, one case investigation regarding exercise with shouting in the fitness facility reported a high attack rate [20]. Similarly, activities such as singing or speaking loudly increase aerosol emissions, thereby increasing the risk of respiratory infectious disease outbreaks [68, 71]. Hence, such activities would benefit from improved ventilation and being moved to an outdoor setting.

This review also collected data on outbreaks in various team sports [29–39]. In team sports, the most common cause of outbreaks was interactions outside of the exercise with inadequate NPIs (e.g. birthday parties, private dinners, buffet lunches, bus transportation and photo sessions). Previous studies analysing game videos and tracking devices reported a low risk of transmission during games, even in high-contact team sports, such as soccer, rugby, and American Football [54, 59, 72, 73]. The outbreak case reports involving baseball, soccer and American Football also indicated that some games had been played during the outbreak, but no transmission of COVID-19 to the opposing team occurred [30, 32, 39]. Even in the case of ice hockey, which is a high contact indoor sport where it is difficult to wear a mask and maintain physical distance, and there is a high risk of COVID-19 outbreak [16, 74], one case investigation reported reducing interactions outside of the game, and strict symptom screening could mitigate player-to-player transmission [35]. Thus, several studies have suggested that the risk of transmission during games in team sports (especially outdoor sports) is low [30, 32, 39]. However, sports involve several events and contacts, such as social events, meeting in person, eating with someone and travelling together, that pose a risk of transmission of infection [60, 75]. These results suggest that reducing interactions outside of exercise or ensuring NPIs in these interactions is equally or more important than typical NPIs, such as wearing a mask and ventilation.

Several case investigations have reported outbreaks caused by participation in sports or exercise among individuals with mild symptoms [19, 21, 26–28, 36]. Periodic laboratory testing is one of the effective measures in mitigating COVID-19 outbreak because patients with COVID-19 often experience mild symptoms or are asymptomatic [17, 29]. However, periodic testing is costly, and self-reported symptom screening often replaces it in recreational and amateur sports. Because self-reported symptom screenings sometimes miss mildly symptomatic individuals [16, 21], clear criteria and participant cooperation are necessary to conduct effective screenings [35].

Individual sports/exercise provide fewer opportunities for human contact than team sports. Individual exercise in an outdoor setting is associated with a low risk of outbreaks [16, 76, 77]. However, Qi et al. reported an outbreak of individuals exercising in an outdoor park [28]. This case investigation revealed that although the attack rate was low, the possibility of outbreaks could not be completely eliminated, even when individuals were exercising in outdoor settings.

No case investigations have reported poor hand hygiene as the main cause of outbreaks. Three papers reported poor surface disinfection as the main cause, without describing the rationale for the cause [25, 27, 32]. Transmission through the surface of an object is known as indirect contact transmission [78]. In a laboratory-based setting, the COVID-19 virus can be detected on sports equipment and in the surrounding environment [79, 80]. However, in the real world, it is rare for transmissible (live) severe acute respiratory syndrome coronavirus 2 (SARS-COV-2) to be detected on environmental surfaces [81–83]. As of 2023, indirect contact transmission is not considered the main mode of transmission of respiratory viruses, such as COVID-19 [63, 83]. This result confirms previous findings that contact transmission is not an important factor in respiratory infection outbreaks, even during sports activities [80].

### Limitations

This study has some limitations. First, all the evidence presented in this study was based on epidemiological case investigations of outbreaks, posing a serious risk of imprecision due to the small number of participants and potential biases in the assessment of exposures or outcomes or both [68]. Second, we did not include news information or grey literature. This study did not cover all outbreaks during sports or exercise and was significantly influenced by publication bias. Third, the review could not make inferences about the extent to which each cause contributed to outbreaks because of the methodological limitations of epidemiological case investigation studies. Fourth, the quality assessment used a unique checklist. The validity of this checklist remains unclear. Fifth, several studies included in this review did not describe the rationale for identifying the main cause of the outbreak. The validity of the causes in these studies is unclear. Sixth, this study did not review non-outbreak cases, where outbreaks were expected but did not occur. The inclusion of such studies could offer a more balanced epidemiological perspective by highlighting both risk and protective factors associated with COVID-19 transmission in these settings. Future research should consider incorporating both outbreak and non-outbreak cases to provide a more comprehensive understanding of the causes of outbreaks. Lastly, most outbreaks occurred in the early stages of the pandemic, before the introduction of vaccines. Since COVID-19 vaccination and previous SARS-COV-2 infections have contributed to herd immunity and helped mitigate outbreaks [84], it is unclear how these results would apply to populations with a high level of immunity against infection.

### Future Work and Implications

Physical activity offers numerous health benefits, such as reduced risk of all-cause mortality, cardiovascular diseases and some cancers and improved musculoskeletal health and mental health [85]. Additionally, it has been shown to prevent severe COVID-19 outcomes [6–8], underscoring its importance even during the pandemic. The results of this systematic review suggest the need for reducing inhalation of aerosol (e.g. ventilation), reducing interactions outside of exercise and symptom screening as NPIs during sports and exercise. Further studies, including intervention and experimental studies, are required to clarify whether combined implementation of these NPIs mitigates outbreaks during sports and exercise.

## Conclusions

This systematic review found evidence suggesting that outbreaks during sports and exercise in the early phase of the COVID-19 pandemic were often caused by a lack of NPIs that reduced the inhalation of aerosol in indoor settings, interactions outside of exercise and the participation of individuals with some symptoms. Ventilation and other NPIs that focus on reducing transmission by inhalation may be important for reducing transmission in indoor facilities. Reducing interactions outside of exercise and NPIs targeting these interactions may be needed to reduce transmission associated with team sports. We found that contact transmission during sports and exercise may not be an important factor in COVID-19 outbreaks. Ventilation, reducing interactions outside of the game, ensuring NPIs in these interactions and symptom screening may be effective preventive strategies in locations where consistent and correct mask wearing and physical distancing are difficult to enforce. These findings could help exercise and sports prepare for COVID-19 and future respiratory infectious disease outbreaks comparable to COVID-19.

## Supplementary Information

Below is the link to the electronic supplementary material.

Supplementary file1 (DOCX 38 KB)

Supplementary file2 (DOCX 62 KB)

## Declarations

### Data availability statement

The data are from published research and, therefore, are in the public domain. The template data collection forms and all data extracted from the included studies are shown in Supplementary Information File 2.
