# Supplementary material for: Causes of COVID-19 Outbreaks During Sports and Exercise: A Systematic Review
Source: Sports Med. 2024 Dec 11;55(3):713–27. doi: 10.1007/s40279-024-02153-7 (PMC11985651; doi:10.1007/s40279-024-02153-7)
Supplement: Supplementary file 1 — Supplementary file1 (DOCX 38 KB) [file 40279_2024_2153_MOESM1_ESM.docx]

Supplementary file 1

**Contents**

Supplementary Table S1. Details of the literature search ･････････････････････････････････････ 2

Supplementary Table S2. Bias assessment tool for evaluating the evidence of severe acute respiratory syndrome coronavirus 2 transmission during sports and exercise ･･････････････････････････････ 7

Supplementary Table S3. Infection prevention strategies of included studies ････････････････････ 8

Supplementary Table S4. Quality assessment of included studies ･････････････････････････････ 9

Supplementary Table S1. Details of the literature search

PubMed

| 1 | (Coronavirus[mh] OR coronavirus[tiab] OR "Coronavirus Infections"[mh] OR "SARS-COVID-2"[tiab] OR "SARS-CoV-2"[tiab] OR "SARS-CoV-2"[mh] OR COVID-19[tiab] OR COVID-19[mh]) |
| --- | --- |
| 2 | ("Disease Transmission, Infectious"[mh] OR transmission[tiab] OR transmissibility[tiab] OR infectiousness [tiab] OR "attack rate"[tiab] OR "Contact Tracing"[mh] OR "contact tracing"[tiab] OR (contacts[tiab] AND tracing[tiab]) OR "active case finding"[tiab] OR "case tracking"[tiab] OR (contact*[tiab] AND investigation[tiab]) OR "disease outbreaks"[mh] OR outbreak*[tiab] OR cluster*[tiab]) |
| 3 | (Sports[mh] OR sports[tiab] OR sport*[tiab] OR Baseball[mh] OR baseball[tiab] OR Basketball[mh] OR Basketball[tiab] OR Bicycling[mh] OR bicycling[tiab] OR Boxing[mh] OR Boxing[tiab] OR "Cricket Sport" [mh] OR "cricket Sport"[tiab] OR Football[mh] OR football[tiab] OR Golf[mh] OR golf[tiab] OR Gymnastics[mh] OR Gymnastics[tiab] OR gym[tiab] OR Hockey[mh] OR hockey[tiab] OR "Martial Arts"[mh] OR "martial arts"[tiab] OR "Tai Ji"[mh] OR "tai ji"[tiab] OR yoga[tiab] OR Mountaineering[mh] OR mountaineering[tiab] OR Tennis[mh] OR tennis[tiab] OR Rugby[mh] OR rugby[tiab] OR Running[mh] OR running[tiab] OR Jogging[mh] OR jogg*[tiab] OR "Marathon Running"[mh] OR "marathon running"[tiab] OR Skating[mh] OR skating[tiab] OR Skiing[mh] OR skiing[tiab] OR Soccer[mh] OR soccer[tiab] OR "Team Sports"[mh] OR "team sports"[tiab] OR Volleyball[mh] OR volleyball[tiab] OR Walking[mh] OR walking[tiab] OR Swimming[mh] OR swimming[tiab] OR swim*[tiab] OR "Weight Lifting"[mh] OR "weight lifting"[tiab] OR Wrestling[mh] OR wrestling[tiab] OR Exercise[mh] OR "Fitness centers"[mh] OR fitness[tiab] OR Dancing[mh] OR dance[tiab] OR danc*[tiab] OR Athletes[mh] OR athlete*[tiab]) |
| 4. | #1 AND #2 AND #3 |

CINAHL（EBSCO）

| 1 | MH Coronavirus OR TI coronavirus OR AB coronavirus OR TI SARS-COVID-2 OR AB SARS-COVID-2 OR TI SARS-CoV-2 OR AB SARS-CoV-2 OR MH SARS-CoV-2 OR TI COVID-19 OR AB COVID-19 OR MH COVID-19 |
| --- | --- |
| 2 | MH Disease Transmission OR TI transmission OR AB transmission OR TI transmissibility OR AB transmissibility OR TI infectiousness OR AB infectiousness OR TI "attack rate" OR AB "attack rate" OR MH contact tracing OR TI "contact tracing" OR AB "contact tracing" OR (TI contacts AND TI tracing) OR (AB contacts AND AB tracing) OR TI "active case finding" OR AB "active case finding" OR TI "case tracking" OR AB "case tracking" OR (TI contact* AND TI investigation) OR (AB contact* AND AB investigation) OR MH disease outbreaks OR TI outbreak* OR AB outbreak* OR TI cluster OR AB cluster OR TI cluster* OR AB cluster* |
| 3 | TI sports OR AB sports OR TI sport* OR AB sport* OR TI baseball OR AB baseball OR TI basketball OR AB basketball OR TI bicycling OR AB bicycling OR TI boxing OR AB boxing OR TI "cricket Sport" OR AB "cricket Sport" OR TI football OR AB football OR TI golf OR AB golf OR TI gymnastics OR AB gymnastics OR TI gym OR AB gym OR TI hockey OR AB hockey OR TI "martial arts" OR AB "martial arts" OR TI "tai ji" OR AB "tai ji" OR TI yoga OR AB yoga OR TI mountaineering OR AB mountaineering OR TI tennis OR AB tennis OR TI rugby OR AB rugby OR TI running OR AB running OR TI jog* OR AB jog* OR TI skating OR AB skating OR TI skiing OR AB skiing OR TI soccer OR AB soccer OR TI "team sports" OR AB "team sports" OR TI volleyball OR AB volleyball OR TI walking OR AB walking OR TI swimming OR AB swimming OR TI swim* OR AB swim* TI "weight lifting" OR AB "weight lifting" OR TI wrestling OR AB wrestling OR TI exercise OR AB exercise OR TI fitness OR AB fitness OR TI dance OR AB dance OR TI danc* OR AB danc* OR TI athlete* OR AB athlete* OR MH Sports OR MH Aeronautical Sports OR MH Amateur Sports OR MH Animal Sports OR MH Fishing OR MH Horseback Riding OR MH Hunting OR MH Aquatic Sports OR MH Diving OR MH Scuba Diving OR MH Snorkeling OR MH Rowing OR MH Swimming OR MH Water Skiing OR MH Athletic Performance OR MH Athletic Training OR MH Athletic Training Programs OR MH Sport Specific Training OR MH Body Building OR MH Bowling OR MH Caving OR MH College Sports OR MH Contact Sports OR MH Boxing OR MH Football OR MH Martial Arts OR MH Rugby OR MH Wrestling OR MH Cycling OR MH Endurance Sports OR MH Extreme Sports OR MH Fencing OR MH Golf OR MH Gymnastics OR MH Handball OR MH Motor Sports OR MH Mountaineering OR MH Professional Sports OR MH Race Walking OR MH Racquet Sports OR MH Tennis OR MH Rock Climbing OR MH Running OR MH Jogging OR MH Running, Distance OR MH Sprinting OR MH Skating OR MH Ice Skating OR MH Skateboarding OR MH Skiing OR MH Snow Skiing OR MH Cross Country Skiing OR MH Water Skiing OR MH Sporting Events OR MH Sports Facilities OR MH Sports for Persons with Disabilities OR MH Wheelchair Sports OR MH Sports Participation OR MH sports re-entry OR MH Talent Identification, sports OR MH Target Sports OR MH Archery OR MH Team Sports OR MH Australian Football OR MH Baseball OR MH Basketball OR MH Cricket (Sports) OR MH Football OR MH Hockey OR MH Rugby OR MH Soccer OR MH Softball OR MH Volleyball OR MH Track and Field OR MH Triathlon OR MH Weight Lifting OR MH Winter Sports OR MH Hockey OR MH Ice Skating OR MH Snow Skiing OR MH Cross Country Skiing OR MH Snowboarding OR MH Exercise OR MH Abdominal Exercises OR MH Aerobic Exercises OR MH Aerobic Dancing OR MH Aquatic Exercises OR MH Jumping OR MH Running OR MH Jogging OR MH Sprinting OR MH Walking OR MH Nordic Walking OR MH Anaerobic Exercises OR MH Back Exercises OR MH Blood Flow Restriction Training OR MH Callisthenics OR MH Core Exercises OR MH Endurance Training OR MH Group Exercise OR MH High-Intensity Interval Training OR MH Lower Extremity Exercises OR MH Dancing OR MH Aerobic Dancing OR MH Ballet OR MH Yoga OR MH Tai Chi OR MH Fitness Centers OR MH Physical Fitness OR MH Personal Training OR MH Athletes |
| 4. | S1 AND S2 AND S3 |

WHO COVID-19 Research Database

| 1 | (("Disease Transmission, Infectious" OR ti:(transmission) OR ab:(transmission) OR ti:(transmissibility) OR ab:(transmissibility) OR ti:(infectiousness) OR ab:(infectiousness) OR ti:(“attack rate”) OR ab:(“attack rate”) OR "Contact Tracing" OR ti:("contact tracing") OR ab:("contact tracing") OR (ti:(contacts) AND ti:(tracing)) OR (ab:(contacts) AND ab:(tracing)) OR ti:("active case finding") OR ab:("active case finding") OR ti:("case tracking") OR ab:("case tracking") OR (ti:(contact*) AND ti:(investigation)) OR (ab:(contact*) AND ab:(investigation)) OR "disease outbreaks" OR ti:(outbreak*) OR ab:(outbreak*) OR ti:(cluster) OR ab:(cluster) OR ti:(cluster*) OR ab:(Cluster*) )) AND (((Sports OR ti:(sports) OR ab:(sports) OR ti:(sport*) OR ab:(sport*) OR Baseball OR ti:(baseball) OR ab:(baseball) OR Basketball OR ti:(basketball) OR ab:(basketball) OR Bicycling OR ti:(bicycling) OR ab:(bicycling) OR Boxing OR ti:(boxing) OR ab:(boxing) OR "Cricket Sport" OR ti:("cricket Sport") OR ab:("cricket Sport") OR Football OR ti:(football) OR ab:(football) OR Golf OR ti:(golf) OR ab:(golf) OR Gymnastics OR ti:(gymnastics) OR ab:(gymnastics) OR ti:(gym) OR ab:(gym) OR Hockey OR ti:(hockey) OR ab:(hockey) OR "Martial Arts" OR ti:("martial arts") OR ab:("martial arts") OR "Tai Ji" OR ti:("tai ji") OR ab:("tai ji") OR ti:(yoga) OR ab:(yoga) OR Mountaineering OR ti:(mountaineering) OR ab:(mountaineering) OR Tennis OR ti:(tennis) OR ab:(tennis) OR Rugby OR ti:(rugby) OR ab:(rugby) OR Running OR ti:(running) OR ab:(running) OR Jogging OR ti:(jog*) OR ab:(jog*) OR "Marathon Running" OR ti:("marathon running") OR ab:("marathon running") OR Skating OR ti:(skating) OR ab:(skating) OR Skiing OR ti:(skiing) OR ab:(skiing) OR Soccer OR ti:(soccer) OR ab:(soccer) OR "Team Sports" OR ti:("team sports") OR ab:("team sports") OR Volleyball OR ti:(volleyball) OR ab:(volleyball) OR Walking OR ti:(walking) OR ab:(walking) OR Swimming OR ti:(swimming) OR ab:(swimming) OR "Weight Lifting" OR ti:("weight lifting") OR ab:("weight lifting") OR Wrestling OR ti:(wrestling) OR ab:(wrestling) OR Exercise OR "Fitness centers" OR ti:(fitness) OR ab:(fitness) OR Dancing OR ti:(dance) OR ab:(dance) OR Athletes OR ti:(athlete*) OR ab:(athlete*))) ) |
| --- | --- |

ICHUSHI web

| 1 | (SARSコロナウイルス-2/TH or COVID-19/TH or COVID-19/AL or (コロナ/TA and DT=2020:2023)) |
| --- | --- |
| 2 | (疾病の発生/TH or 疾病の流行/TH or 疾病多発地域/TH or (集団発生/TI or 集団発生/AB) or (アウトブレイク/TI or アウトブレイク/AB) or (アウトブレーク/TI or アウトブレーク/AB) or (クラスター/TI or クラスター/AB) or (集団感染/TI or 集団感染/AB) or (集団発生/TI or 集団発生/AB) or (患者発生/TI or 患者発生/AB) or 接触歴/TH or (接触歴/TI or 接触歴/AB) or 接触者/TH or (接触者/TI or 接触者/AB) or (濃厚接触/TI or 濃厚接触/AB) or 実態調査/TH or (実態調査/TI or 実態調査/AB) or 感染症伝播/TH or クラスター発生地域/TH) |
| 3 | (スポーツ/TH or (スポーツ/TI or スポーツ/AB) or 野球/TH or (野球/TI or 野球/AB) or バスケットボール/TH or (バスケットボール/TI or バスケットボール/AB) or 自転車/TH or (自転車/TI or 自転車/AB) or ボクシング/TH or (ボクシング/TI or ボクシング/AB) or 球技スポーツ/TH or クリケット/TH or (クリケット/TI or クリケット/AB) or フットボール/TH or (フットボール/TI or フットボール/AB) or ゴルフ/TH or (ゴルフ/TI or ゴルフ/AB) or 体操/TH or (体操/TI or 体操/AB) or (ジム/TI or ジム/AB) or ホッケー/TH or (ホッケー/TI or ホッケー/AB) or 武道/TH or (武道/TI or 武道/AB) or 合気道/TH or (合気道/TI or 合気道/AB) or 空手/TH or (空手/TI or 空手/AB) or 剣道/TH or (剣道/TI or 剣道/AB) or 柔道/TH or (柔道/TI or 柔道/AB) or 相撲/TH or (相撲/TI or 相撲/AB) or 太極拳/TH or (太極拳/TI or 太極拳/AB) or ヨガ/TH or (ヨガ/TI or ヨガ/AB) or 登山/TH or (登山/TI or 登山/AB) or ラケットスポーツ/TH or (ラケットスポーツ/TI or ラケットスポーツ/AB) or 卓球/TH or (卓球/TI or 卓球/AB) or バドミントン/TH or (バドミントン/TI or バドミントン/AB) or テニス/TH or (テニス/TI or テニス/AB) or ラグビー/TH or (ラグビー/TI or ラグビー/AB) or ランニング/TH or (ランニング/TI or ランニング/AB) or ジョギング/TH or (ジョギング/TI or ジョギング/AB) or マラソン/TH or (マラソン/TI or マラソン/AB) or スケート/TH or (スケート/TI or スケート/AB) or スキー/TH or (スキー/TI or スキー/AB) or サッカー/TH or (サッカー/TI or サッカー/AB) or チームスポーツ/TH or (チームスポーツ/TI or チームスポーツ/AB) or バレーボール/TH or (バレーボール/TI or バレーボール/AB) or 歩行/TH or (歩行/TI or 歩行/AB) or 水泳/TH or (水泳/TI or 水泳/AB) or 重量挙げ/TH or (重量挙げ/TI or 重量挙げ/AB) or レスリング/TH or (レスリング/TI or レスリング/AB) or 身体運動/TH or (エクササイズ/TI or エクササイズ/AB) or (運動/TI or 運動/TI) or 健康増進センター/TH or (フィットネス/TI or フィットネス/AB) or 舞踏/TH or (ダンス/TI or ダンス/AB) or 陸上競技/TH or (陸上競技/TI or 陸上競技/AB) or 運動選手/TH or (運動選手/TI or 運動選手/AB or アスリート/TI or アスリート/AB or スポーツ選手/TI or スポーツ選手/AB or 競技者/AB or 競技者/TI) or 障害者運動選手/TH) |
| 4 | #1 and #2 and #3 |

Supplementary Table S2. Bias assessment tool for evaluating the evidence of severe acute respiratory syndrome coronavirus 2 transmission during sports and exercise

| Criteria | Points awarded or withdrawn |
| --- | --- |
| Index case classification |  |
| Laboratory confirmation | 1 |
| Unspecific clinical presentation or data not provided | 0 |
| Secondary case ascertainment |  |
| Laboratory confirmation of all cases | 2 |
| Syndromic or no comprehensive confirmation of all secondary cases | 1 |
| Not provided | 0 |
| Case investigation strategy |  |
| Comprehensive | 2 |
| Others | 0 |
| Timeliness of case investigation |  |
| Within 1 week | 2 |
| Within 3 weeks | 1 |
| After 3 weeks or more/not provided | 0 |
| Completeness: proportion of participants followed up |  |
| More than 80% were followed up | 2 |
| Between 80% and 50% were followed up | 1 |
| Less than 50% were followed up/not provided | 0 |
| Limitations |  |
| Exclude possible alternative transmission (e.g. genomic sequencing) | 0 |
| Not exclude possible alternative transmission (e.g. no genomic sequencing) | -1 |

Resulting evidence levels: 0–3, low; 4–6, medium; 7–9, high

The original checklist was adapted from a checklist used in a previous systematic review of influenza transmission in aircraft.^47^

Supplementary Table S3. Infection prevention strategies of included studies

| First author | Type of sports | Wearing mask during exercise | Wearing mask outside of exercise | Maintaining physical distance during exercise | Maintaining physical distance during outside of exercise | Ventilation | Hand hygiene | Surface  disinfection | Bubble protocol | Limitations on capacity | Temperature check | Physical condition self-check | Periodic laboratory screening | Vaccination |
| --- | --- | --- | --- | --- | --- | --- | --- | --- | --- | --- | --- | --- | --- | --- |
| Jang S | fitness class | NP | NP | N | NP | NP | NP | NP | NP | NP | NP | NP | NP | NP |
| Groves LM | fitness class | N | NP | N | NP | N | NP | NP | NP | NP | NP | NP | NP | NP |
| Lendacki FR | fitness class | N | Y | Y | NP | N | NP | NP | NP | Y | Y | Y | NP | NP |
| Bart SM | fitness class | N | Y | NP | NP | NP | NP | NP | NP | NP | NP | NP | NP | NP |
| Chu DKW | fitness class | N | NP | NP | NP | N | NP | NP | NP | NP | NP | NP | NP | N |
| Anderson M | workout | N | N | N | N | Y | NP | Y | NP | NP | NP | NP | NP | NP |
| Brlek A | squash | NP | NP | NP | NP | N | NP | NP | NP | NP | NP | NP | NP | NP |
| Shin SH | taekwondo class | N | N | NP | NP | Y | Y | NP | NP | NP | Y | N | NP | N |
| Dougherty K | gymnastics class | N | N | NP | NP | N | NP | N | NP | NP | NP | NP | NP | N |
| Qi L | jogging | N | NP | N | NP | NP | NP | NP | NP | NP | NP | NP | NP | NP |
| Teran RA | soccer | Y | N | NP | N | NP | NP | NP | NP | NP | NP | NP | NP | NP |
| Schumacher YO | soccer | N | Y | N | Y | NP | Y | NP | N | NP | Y | Y | Y | NP |
| Basu S | soccer | N | N | N | Y | NP | Y | Y | NP | NP | Y | Y | Y | NP |
| Siegel M | American football | N | NP | NP | N | N | NP | N | NP | NP | NP | NP | NP | NP |
| Mack CD | American football | NP | NP | N | Y | NP | Y | Y | NP | Y | NP | NP | Y | NP |
| Atrubin D | ice hockey | N | N | N | N | NP | NP | NP | NP | NP | NP | NP | NP | NP |
| Krug A | ice hockey | N | Y | N | Y | NP | NP | NP | NP | N | Y | Y | N | NP |
| Burak KW | curling | NP | NP | NP | NP | NP | Y | Y | NP | Y | NP | NP | NP | NP |
| Morath O | volleyball | N | Y | NP | NP | NP | NP | NP | NP | NP | NP | NP | Y | NP |
| Pauser J | basketball | N | N | N | Y | NP | NP | NP | NP | Y | Y | Y | N | NP |
| Murray MT | baseball | N | Y | N | Y | NP | NP | Y | N | NP | Y | Y | Y | NP |

Y: Yes, N: No, NP: Not provided

Supplementary Table S4. Quality assessment of included studies

| First author | Index case classification | Secondary case ascertainment | Case investigation strategy | Timeliness of case investigation | Completeness | Limitations | Score | Evidence level |
| --- | --- | --- | --- | --- | --- | --- | --- | --- |
| Jang S | 1 | 2 | 0 | 0 | 0 | -1 | 2 | Low |
| Groves LM | 1 | 2 | 2 | 2 | 2 | -1 | 8 | High |
| Lendacki FR | 1 | 2 | 0 | 0 | 2 | -1 | 4 | Medium |
| Bart SM | 1 | 2 | 0 | 0 | 0 | 0 | 3 | Low |
| Chu DKW | 1 | 2 | 2 | 1 | 2 | 0 | 8 | High |
| Anderson M | 1 | 2 | 2 | 2 | 0 | 0 | 7 | High |
| Brlek A | 1 | 2 | 2 | 0 | 2 | -1 | 6 | Medium |
| Shin SH | 1 | 2 | 0 | 1 | 2 | -1 | 5 | Medium |
| Dougherty K | 0 | 1 | 2 | 0 | 2 | 0 | 5 | Medium |
| Qi L | 1 | 2 | 2 | 2 | 2 | 0 | 9 | High |
| Teran RA | 1 | 2 | 2 | 0 | 2 | 0 | 7 | High |
| Schumacher YO | 1 | 2 | 2 | 0 | 2 | -1 | 6 | Medium |
| Basu S | 1 | 2 | 2 | 0 | 2 | -1 | 6 | Medium |
| Siegel M | 1 | 2 | 2 | 0 | 0 | -1 | 4 | Medium |
| Mack CD | 1 | 2 | 2 | 2 | 2 | -1 | 8 | High |
| Atrubin D | 1 | 1 | 2 | 2 | 2 | -1 | 7 | High |
| Krug A | 1 | 2 | 0 | 0 | 0 | -1 | 2 | Low |
| Burak KW | 0 | 1 | 2 | 0 | 2 | -1 | 4 | Medium |
| Morath O | 1 | 2 | 2 | 1 | 2 | -1 | 7 | High |
| Pauser J | 1 | 2 | 0 | 0 | 2 | -1 | 4 | Medium |
| Murray MT | 1 | 2 | 2 | 2 | 2 | 0 | 9 | High |
