# Supplementary material for: Causes of COVID-19 Outbreaks During Sports and Exercise: A Systematic Review
Source: Sports Med. 2024 Dec 11;55(3):713–27. doi: 10.1007/s40279-024-02153-7 (PMC11985651; doi:10.1007/s40279-024-02153-7)
Supplement: Supplementary file 2 — Supplementary file2 (DOCX 62 KB) [file 40279_2024_2153_MOESM2_ESM.docx]

Supplementary file 2. Template data collection forms and data extracted from included studies

**Contents**

1. Characteristics ･･････････････････････････････････････････････････････････････････････････････････････････････････････ 2
2. Preventive strategies ･･････････････････････････････････････････････････････････････････････････････････････････････････ 7
3. Details of case investing ･･････････････････････････････････････････････････････････････････････････････････････････････ 11
4. Bias Assessment ････････････････････････････････････････････････････････････････････････････････････････････････････ 20

1. Characteristics

| Title | First Author | Category | Type of sports | Location | Date | Setting | Purpose | Unit | Duration and Frequency | Number of participants | Age [median (range)] | Sex [women n(%)] | Note |
| --- | --- | --- | --- | --- | --- | --- | --- | --- | --- | --- | --- | --- | --- |
| Cluster of Coronavirus Disease Associated with Fitness Dance Classes, South Korea | Jang S | Individual sports or exercise | fitness class | South Korea | 2020.2-3 | fitness facility | leisure | group | 50min/time, 2times/week | 217 | NP | NP | The number is only students. Number of sports-related exposures including instructors is unknown. |
| Community Transmission of SARS-CoV-2 at Three Fitness Facilities - Hawaii, June-July 2020 | Groves LM | Individual sports or exercise | fitness class | America | 2020.6-7 | fitness facility | leisure | group | NP | 62 | NP | NP | - |
| COVID-19 Outbreak Among Attendees of an Exercise Facility - Chicago, Illinois, August-September 2020 | Lendacki FR | Individual sports or exercise | fitness class | America | 2020.8-9 | fitness facility | leisure | group | NP | 81 | 42(NP) | 71 (87.7) | Excluded 10 people who had no data (91 total participants) |
| Multiple Transmission Chains within COVID-19 Cluster, Connecticut, USA, 2020^1^ | Bart SM | Individual sports or exercise | fitness class | America | 2020 | fitness facility | leisure | group | 60min/time, 1 time | 7 | NP | NP | Collected data about fitness class outbreak  Date unknown (fall) 1 positive instructor + approximately 6 participants |
| SARS-CoV-2 Superspread in Fitness Center, Hong Kong, China, March 2021 | Chu DKW | Individual sports or exercise | fitness class | China | 2021.2-3 | fitness facility | leisure | group | NP | 301 | NP | NP | approximately 300 visitors + one index case |
| An outbreak of COVID-19 associated with a fitness centre in Saskatchewan: Lessons for prevention | Anderson M | Individual sports or exercise | workout | Canada | 2020.9-10 | fitness facility | leisure | individual | NP | 251 | NP | NP | - |
| Possible indirect transmission of COVID-19 at a squash court, Slovenia, March 2020: case report | Brlek A | Individual sports or exercise | squash | Slovenia | 2020.3 | Squash court | leisure | one on one | 45-60min/time, 3 times | 6 | NP | NP | There were five other employees at the facility who were not symptomatic. |
| COVID-19 outbreak and risk factors for infection in a taekwondo gym in the Republic of Korea | Shin SH | Individual sports or exercise | taekwondo class | South Korea | 2021.1-2 | taekwondo gym | leisure | group | 50min/time | 108 | NP | NP | There are participants characteristics for 92 respondents, but none for 108. |
| SARS-CoV-2 B.1.617.2 (Delta) Variant COVID-19 Outbreak Associated with a Gymnastics Facility - Oklahoma, April-May 2021 | Dougherty K | Individual sports or exercise | gymnastics class | America | 2021.4-5 | gymnastics facilities | leisure | group | NP | 133 | NP | NP | - |
| An Outbreak of SARS-CoV-2 Omicron Subvariant BA.2.76 in an Outdoor Park - Chongqing Municipality, China, August 2022 | Qi L | Individual sports or exercise | jogging | China | 2022.8 | outdoor park | leisure | individual | 35min/time, 1time | 2836 | NP | NP | - |
| COVID-19 Outbreak Among a University's Men's and Women's Soccer Teams - Chicago, Illinois, July-August 2020 | Teran RA | Team sports | soccer | America | 2020.7-8 | Voluntary training session | university team | group | NP | 45 | 20(NP) | 24(53.3) | - |
| Resuming professional football (soccer) during the COVID-19 pandemic in a country with high infection rates: a prospective cohort study | Schumacher YO | Team sports | soccer | Qatar | 2020 | Professional Sports Season | professional team | group | NP | NP | NP | NP | Collected data on two outbreak that matched the definition of an outbreak in this study  Date unknown |
| Surveillance for COVID-19 in the English Football League 2019-2020 | Basu S | Team sports | soccer | United Kingdom | 2020 | Professional Sports Season | professional team | group | NP | NP | NP | NP | Collected data on one outbreak that matched the definition of an outbreak in this study  Date unknown |
| Notes from the Field: SARS-CoV-2 Transmission Associated with High School Football Team Members - Florida, September-October 2020 | Siegel M | Team sports | American football | America | 2020.9-10 | School environment | school team | group | NP | 54 | NP | NP | High school team |
| Implementation and Evolution of Mitigation Measures, Testing, and Contact Tracing in the National Football League, August 9-November 21, 2020 | Mack CD | Team sports | American football | America | 2020.9-10 | Professional Sports Season | professional team | group | NP | NP | NP | NP | collected a data about one outbreak case from September 27 to October 10, 2011 |
| An Outbreak of COVID-19 Associated with a Recreational Hockey Game - Florida, June 2020 | Atrubin D | Team sports | ice hockey | America | 2020.6 | indoor ice rink | leisure | group | 60min/time, 1time | 22 | (19-53) | 0 | excluded rink staff |
| Youth ice hockey COVID-19 protocols and prevention of sport-related transmission | Krug A | Team sports | ice hockey | America | 2020.10-11 | ice rink | youth team | group | NP | NP | NP | NP | Collected data on one outbreak that matched the definition of an outbreak in this study |
| COVID-19 outbreak among physicians at a Canadian curling bonspiel: a descriptive observational study | Burak KW | Team sports | curling | Canada | 2020.3 | curling rink | leisure | group | NP | 73 | 51(26-79) | 18 (24.7%) | - |
| Case Report: Assessing COVID-19 Transmission in Professional Volleyball in Germany, September to December 2020: An Epidemiological Study | Morath O | Team sports | volleyball | Germany | 2020.9 | Professional Sports Season | professional team | group | NP | 15 | NP | NP | Player age range was 18-33 (15 participants includes staff and player) |
| SARS-CoV-2 transmission during an indoor professional sporting event | Pauser J | Team sports | basketball | Germany | 2020.11 | indoor basket court | professional team | group | NP | 69 | NP | NP | - |
| Mitigating a COVID-19 Outbreak Among Major League Baseball Players - United States, 2020 | Murray MT | Team sports | baseball | America | 2020 | Professional Sports Season | professional team | group | NP | 146 | NP | NP | Date unknown, total infectious persons play time 40hours 23min |

NP: Not provided

2. Preventive strategies

| Title | First author | Category | Type of sports | Wearing mask during exercise | Wearing mask outside of exercise | Maintaining physical distance during exercise | Maintaining physical distance during outside of exercise | Ventilation | Hand hygiene | Surface disinfection | Bubble protocol | Limitations on capacity | Temperature check | Physical condition self-check | Periodic laboratory screening | Vaccination | Note |
| --- | --- | --- | --- | --- | --- | --- | --- | --- | --- | --- | --- | --- | --- | --- | --- | --- | --- |
| Cluster of Coronavirus Disease Associated with Fitness Dance Classes, South Korea | Jang S | Individual sports or exercise | fitness class | NP | NP | N | NP | NP | NP | NP | NP | NP | NP | NP | NP | NP | small space, and intensity of the workouts |
| Community Transmission of SARS-CoV-2 at Three Fitness Facilities - Hawaii, June-July 2020 | Groves LM | Individual sports or exercise | fitness class | N | NP | N | NP | N | NP | NP | NP | NP | NP | NP | NP | NP | maintain: cycling class: yes kick boxing class: no |
| COVID-19 Outbreak Among Attendees of an Exercise Facility - Chicago, Illinois, August-September 2020 | Lendacki FR | Individual sports or exercise | fitness class | N | Y | Y | NP | N | NP | NP | NP | Y | Y | Y | NP | NP | - |
| Multiple Transmission Chains within COVID-19 Cluster, Connecticut, USA, 2020^1^ | Bart SM | Individual sports or exercise | fitness class | N | Y | NP | NP | NP | NP | NP | NP | NP | NP | NP | NP | NP | - |
| SARS-CoV-2 Superspread in Fitness Center, Hong Kong, China, March 2021 | Chu DKW | Individual sports or exercise | fitness class | N | NP | NP | NP | N | NP | NP | NP | NP | NP | NP | NP | N | - |
| An outbreak of COVID-19 associated with a fitness centre in Saskatchewan: Lessons for prevention | Anderson M | Individual sports or exercise | workout | N | N | N | N | Y | NP | Y | NP | NP | NP | NP | NP | NP | There were regulations regarding surface disinfection and ventilation, but they did not evaluate what they were. |
| Possible indirect transmission of COVID-19 at a squash court, Slovenia, March 2020: case report | Brlek A | Individual sports or exercise | squash | NP | NP | NP | NP | N | NP | NP | NP | NP | NP | NP | NP | NP | - |
| COVID-19 outbreak and risk factors for infection in a taekwondo gym in the Republic of Korea | Shin SH | Individual sports or exercise | taekwondo class | N | N | NP | NP | Y | Y | NP | NP | NP | Y | N | NP | N | - |
| SARS-CoV-2 B.1.617.2 (Delta) Variant COVID-19 Outbreak Associated with a Gymnastics Facility - Oklahoma, April-May 2021 | Dougherty K | Individual sports or exercise | gymnastics class | N | N | NP | NP | N | NP | N | NP | NP | NP | NP | NP | N | - |
| An Outbreak of SARS-CoV-2 Omicron Subvariant BA.2.76 in an Outdoor Park - Chongqing Municipality, China, August 2022 | Qi L | Individual sports or exercise | jogging | N | NP | N | NP | NP | NP | NP | NP | NP | NP | NP | NP | NP | - |
| COVID-19 Outbreak Among a University's Men's and Women's Soccer Teams - Chicago, Illinois, July-August 2020 | Teran RA | Team sports | soccer | Y | N | NP | N | NP | NP | NP | NP | NP | NP | NP | NP | NP | - |
| Resuming professional football (soccer) during the COVID-19 pandemic in a country with high infection rates: a prospective cohort study | Schumacher YO | Team sports | soccer | N | Y | N | Y | NP | Y | NP | N | NP | Y | Y | Y | NP | - |
| Surveillance for COVID-19 in the English Football League 2019-2020 | Basu S | Team sports | soccer | N | N | N | Y | NP | Y | Y | NP | NP | Y | Y | Y | NP | - |
| Notes from the Field: SARS-CoV-2 Transmission Associated with High School Football Team Members - Florida, September-October 2020 | Siegel M | Team sports | American football | N | NP | NP | N | N | NP | N | NP | NP | NP | NP | NP | NP | - |
| Implementation and Evolution of Mitigation Measures, Testing, and Contact Tracing in the National Football League, August 9-November 21, 2020 | Mack CD | Team sports | American football | NP | NP | N | Y | NP | Y | Y | NP | Y | NP | NP | Y | NP | Regulations required masking, but there were unmasking meetings. |
| An Outbreak of COVID-19 Associated with a Recreational Hockey Game - Florida, June 2020 | Atrubin D | Team sports | ice hockey | N | N | N | N | NP | NP | NP | NP | NP | NP | NP | NP | NP | - |
| Youth ice hockey COVID-19 protocols and prevention of sport-related transmission | Krug A | Team sports | ice hockey | N | Y | N | Y | NP | NP | NP | NP | N | Y | Y | N | NP | - |
| COVID-19 outbreak among physicians at a Canadian curling bonspiel: a descriptive observational study | Burak KW | Team sports | curling | NP | NP | NP | NP | NP | Y | Y | NP | Y | NP | NP | NP | NP | Limit indoor gatherings to 250 people |
| Case Report: Assessing COVID-19 Transmission in Professional Volleyball in Germany, September to December 2020: An Epidemiological Study | Morath O | Team sports | volleyball | N | Y | NP | NP | NP | NP | NP | NP | NP | NP | NP | Y | NP | - |
| SARS-CoV-2 transmission during an indoor professional sporting event | Pauser J | Team sports | basketball | N | N | N | Y | NP | NP | NP | NP | Y | Y | Y | N | NP | no visitors |
| Mitigating a COVID-19 Outbreak Among Major League Baseball Players - United States, 2020 | Murray MT | Team sports | baseball | N | Y | N | Y | NP | NP | Y | N | NP | Y | Y | Y | NP | - |

Y: Yes, N:No, NP: Not provided

3. Details of case investing

| Title | First author | Category | Type of sports | Case investigation method | Outcome measure | Case ascertainment method | Number of participants | Number of cases | Attack rate | Cause: not wearing mask during exercise | Cause: not wearing mask outside of exercise | Cause: not maintaining physical distance during exercise | Cause: not maintaining physical distance during outside of exercise | Cause: crowded places | Cause: poor ventilation | Cause: insufficient hand hygiene | Cause: poor surface disinfection | Cause: interactions outside of exercise | Cause: participate with some symptoms | Other / note | Rationale for the cause | Note |
| --- | --- | --- | --- | --- | --- | --- | --- | --- | --- | --- | --- | --- | --- | --- | --- | --- | --- | --- | --- | --- | --- | --- |
| Cluster of Coronavirus Disease Associated with Fitness Dance Classes, South Korea | Jang S | Individual sports or exercise | fitness class | NP | attack rate | PCR | 217 | 57 | 26.3% | - | - | - | - | Y | - | - | - | - | - | high intensity exercise | (crowded places) No one infected in a small class. (high intensity exercise) There are no case in Pilates and yoga classes. | Instructors taught class with very mild symptoms, such as coughs. |
| Community Transmission of SARS-CoV-2 at Three Fitness Facilities - Hawaii, June-July 2020 | Groves LM | Individual sports or exercise | fitness class | Interview | attack rate | PCR | 62 | 21 | 33.9% | Y | - | Y | - | - | Y | - | - | - | - | shouting | NP | - |
| COVID-19 Outbreak Among Attendees of an Exercise Facility - Chicago, Illinois, August-September 2020 | Lendacki FR | Individual sports or exercise | fitness class | Interview | attack rate | PCR | 81 | 49 | 60.5% | Y | - | - | - | - | Y | - | - | - | Y | NP | (mask) The odds ratio is high, although not significant. | 7 symptomatic patients besides 49 (not tested or negative) /  Two were participated with symptomatic condition. |
| Multiple Transmission Chains within COVID-19 Cluster, Connecticut, USA, 2020^1^ | Bart SM | Individual sports or exercise | fitness class | Interview | number of cases | PCR | 7 | 3 | 42.9%* | Y | - | - | - | - | - | - | - | - | - | NP | NP | - |
| SARS-CoV-2 Superspread in Fitness Center, Hong Kong, China, March 2021 | Chu DKW | Individual sports or exercise | fitness class | NP | number of cases | PCR | 301 | 102 | 33.9%* | Y | - | - | - | - | Y | - | - | - | - | NP | NP | number of cases: 101 + 1 index case |
| An outbreak of COVID-19 associated with a fitness centre in Saskatchewan: Lessons for prevention | Anderson M | Individual sports or exercise | workout | Interview | attack rate | PCR | 251 | 27 | 10.8% | - | - | - | - | - | Y | - | - | - | - | NP | NP | - |
| Possible indirect transmission of COVID-19 at a squash court, Slovenia, March 2020: case report | Brlek A | Individual sports or exercise | squash | NP | number of cases | PCR | 6 | 5 | 83.3%* | - | - | - | - | - | Y | - | Y | - | - | high intensity exercise | NP | The remaining one is symptomatic but not tested. |
| COVID-19 outbreak and risk factors for infection in a taekwondo gym in the Republic of Korea | Shin SH | Individual sports or exercise | taekwondo class | Questionnaire | attack rate | PCR | 108 | 30 | 27.8% | - | - | - | - | - | - | - | - | Y | Y | (interactions outside of exercise) food consumption inside the gym / teatime | (interactions outside of game) Odds ratio was significantly high. | - |
| SARS-CoV-2 B.1.617.2 (Delta) Variant COVID-19 Outbreak Associated with a Gymnastics Facility - Oklahoma, April-May 2021 | Dougherty K | Individual sports or exercise | gymnastics class | Interview | attack rate | PCR+Symptom | 133 | 26 | 19.5% | Y | - | - | - | - | Y | - | Y | - | Y | low vaccination | NP | Out of 47cases, 23 gym users and 3 staff. The remaining 21 were secondary infection cases. /  delayed recognition of infection because of mild symptoms or attribution of symptoms to other causes. |
| An Outbreak of SARS-CoV-2 Omicron Subvariant BA.2.76 in an Outdoor Park - Chongqing Municipality, China, August 2022 | Qi L | Individual sports or exercise | jogging | Interview | number of cases | PCR | 2836 | 39 | 1.4%* | Y | - | Y | - | - | - | - | - | - | - | NP | (mask) Most of the cases were not wearing masks. (physical distance) There are many positives among those who did not maintain physical distance. | Index case had fatigue while jogging. |
| COVID-19 Outbreak Among a University's Men's and Women's Soccer Teams - Chicago, Illinois, July-August 2020 | Teran RA | Team sports | soccer | Interview | attack rate | PCR | 45 | 17 | 37.8% | - | - | - | - | - | - | - | - | Y | - | (interactions outside of exercise) birthday parties, etc. / Living together in a share house | (interactions outside of game) All seven birthday party attendees are positive. (Living together in a share house) Odds ratio is significantly high. | - |
| Resuming professional football (soccer) during the COVID-19 pandemic in a country with high infection rates: a prospective cohort study | Schumacher YO | Team sports | soccer | NP | number of cases | PCR | NP | 2 / 4 | NP | - | - | - | - | - | - | - | - | Y | - | (interactions outside of exercise) sharing a car / private dinner | (interactions outside of game) No other teammates were infected. / None of other teammates were subsequently found to be infected. | There were two outbreak cases due to interactions outside of game. |
| Surveillance for COVID-19 in the English Football League 2019-2020 | Basu S | Team sports | soccer | NP | number of cases | PCR | NP | 4 | NP | - | - | - | - | - | - | - | - | Y | - | (interactions outside of exercise) contact with an infected individual in the community. | (interactions outside of game)　There were no infected people who had no contact outside the game. | Five infected in one team at the same time, but four outbreak-related. |
| Notes from the Field: SARS-CoV-2 Transmission Associated with High School Football Team Members - Florida, September-October 2020 | Siegel M | Team sports | American football | Interview | number of cases | PCR | 54 | 14 | 25.9%* | Y | - | - | - | - | - | - | Y | Y | - | (interactions outside of exercise) bus transporting | NP | inadequate physical distancing and air ventilation on buses transporting players. |
| Implementation and Evolution of Mitigation Measures, Testing, and Contact Tracing in the National Football League, August 9-November 21, 2020 | Mack CD | Team sports | American football | Interview | number of cases | PCR | NP | 21 | NP | - | Y | - | Y | - | - | - | - | Y | - | (interactions outside of exercise) eating | NP | - |
| An Outbreak of COVID-19 Associated with a Recreational Hockey Game - Florida, June 2020 | Atrubin D | Team sports | ice hockey | Interview | number of cases | PCR+Antigen+Symptom | 22 | 14 | 63.6%* | - | - | Y | Y | - | - | - | - | - | - | NP | NP | - |
| Youth ice hockey COVID-19 protocols and prevention of sport-related transmission | Krug A | Team sports | ice hockey | NP | number of cases | PCR+Antigen | NP | 33 | NP | - | - | - | - | - | - | - | - | Y | - | (interactions outside of exercise) indoor picture night | The peak of the outbreak was confirmed five days after the picture night. | - |
| COVID-19 outbreak among physicians at a Canadian curling bonspiel: a descriptive observational study | Burak KW | Team sports | curling | Questionnaire | attack rate | PCR+Symptom | 73 | 56 | 76.7% | - | - | - | - | - | - | - | - | Y | Y | (interactions outside of exercise) buffet lunches at carling rink, etc. | (interactions outside of game) Significantly more of the positive participants attended the lunch buffet. /  There were no case of the team who did not participate in all social events. | Ten were participated with symptomatic condition. |
| Case Report: Assessing COVID-19 Transmission in Professional Volleyball in Germany, September to December 2020: An Epidemiological Study | Morath O | Team sports | volleyball | NP | number of cases | PCR | 15 | 5 | 33.3%* | - | - | - | - | - | - | - | - | Y | - | (interactions outside of exercise) social gathering in a bar | (interactions outside of game) No one who did not attend the bar event tested positive. | - |
| SARS-CoV-2 transmission during an indoor professional sporting event | Pauser J | Team sports | basketball | NP | number of cases | PCR | 69 | 36 | 52.2％* | Y | Y | - | - | - | - | - | - | - | - | NP | (mask) The number of positive cases is high among those who do not wear masks. | - |
| Mitigating a COVID-19 Outbreak Among Major League Baseball Players - United States, 2020 | Murray MT | Team sports | baseball | NP | attack rate | PCR | 146 | 21 | 14.4% | - | - | - | - | - | - | - | - | Y | - | (interactions outside of exercise) Interactions outside of on-filed play | (interactions outside of game) None of the players on the opposing team were infected. | - |

*: Not described in the manuscript. The authors calculated the attack rate. Y: Yes

4. Bias Assessment

| Title | First author | Category | Index case classification | Secondary case ascertainment | Case investigation strategy | Timeliness of case investigation | Completeness | Limitations | Score | Evidence level | Note |
| --- | --- | --- | --- | --- | --- | --- | --- | --- | --- | --- | --- |
| Cluster of Coronavirus Disease Associated with Fitness Dance Classes, South Korea | Jang S | Individual sports or exercise | 1 | 2 | 0 | 0 | 0 | -1 | 2 | Low | - |
| Community Transmission of SARS-CoV-2 at Three Fitness Facilities - Hawaii, June-July 2020 | Groves LM | Individual sports or exercise | 1 | 2 | 2 | 2 | 2 | -1 | 8 | High | - |
| COVID-19 Outbreak Among Attendees of an Exercise Facility - Chicago, Illinois, August-September 2020 | Lendacki FR | Individual sports or exercise | 1 | 2 | 0 | 0 | 2 | -1 | 4 | Medium | (Strategy) Tracking 81 of 91 patients. Possibly no PCR testing in some. |
| Multiple Transmission Chains within COVID-19 Cluster, Connecticut, USA, 2020^1^ | Bart SM | Individual sports or exercise | 1 | 2 | 0 | 0 | 0 | 0 | 3 | Low | (Timeliness) No date given when the survey was started. (Strategy) Also stated as "approximately", so it is unclear if it is all of them. |
| SARS-CoV-2 Superspread in Fitness Center, Hong Kong, China, March 2021 | Chu DKW | Individual sports or exercise | 1 | 2 | 2 | 1 | 2 | 0 | 8 | High | (Strategy / Completeness) Mandatory testing was described. |
| An outbreak of COVID-19 associated with a fitness centre in Saskatchewan: Lessons for prevention | Anderson M | Individual sports or exercise | 1 | 2 | 2 | 2 | 0 | 0 | 7 | High | - |
| Possible indirect transmission of COVID-19 at a squash court, Slovenia, March 2020: case report | Brlek A | Individual sports or exercise | 1 | 2 | 2 | 0 | 2 | -1 | 6 | Medium | - |
| COVID-19 outbreak and risk factors for infection in a taekwondo gym in the Republic of Korea | Shin SH | Individual sports or exercise | 1 | 2 | 0 | 1 | 2 | -1 | 5 | Medium | (Strategy) 92 respondents out of 108 |
| SARS-CoV-2 B.1.617.2 (Delta) Variant COVID-19 Outbreak Associated with a Gymnastics Facility - Oklahoma, April-May 2021 | Dougherty K | Individual sports or exercise | 0 | 1 | 2 | 0 | 2 | 0 | 5 | Medium | (Strategy) The roster was acquired, and the incidence rate was calculated. |
| An Outbreak of SARS-CoV-2 Omicron Subvariant BA.2.76 in an Outdoor Park - Chongqing Municipality, China, August 2022 | Qi L | Individual sports or exercise | 1 | 2 | 2 | 2 | 2 | 0 | 9 | High | - |
| COVID-19 Outbreak Among a University's Men's and Women's Soccer Teams - Chicago, Illinois, July-August 2020 | Teran RA | Team sports | 1 | 2 | 2 | 0 | 2 | 0 | 7 | High | - |
| Resuming professional football (soccer) during the COVID-19 pandemic in a country with high infection rates: a prospective cohort study | Schumacher YO | Team sports | 1 | 2 | 2 | 0 | 2 | -1 | 6 | Medium | (Strategy / Completeness) It's a professional sport related outbreak, and it could be all countable and traceable. |
| Surveillance for COVID-19 in the English Football League 2019-2020 | Basu S | Team sports | 1 | 2 | 2 | 0 | 2 | -1 | 6 | Medium | (Strategy / Completeness) It's a professional sport related outbreak, and it could be all countable and traceable. |
| Notes from the Field: SARS-CoV-2 Transmission Associated with High School Football Team Members - Florida, September-October 2020 | Siegel M | Team sports | 1 | 2 | 2 | 0 | 0 | -1 | 4 | Medium | (Completeness) No data on 31 people |
| Implementation and Evolution of Mitigation Measures, Testing, and Contact Tracing in the National Football League, August 9-November 21, 2020 | Mack CD | Team sports | 1 | 2 | 2 | 2 | 2 | -1 | 8 | High | - |
| An Outbreak of COVID-19 Associated with a Recreational Hockey Game - Florida, June 2020 | Atrubin D | Team sports | 1 | 1 | 2 | 2 | 2 | -1 | 7 | High | - |
| Youth ice hockey COVID-19 protocols and prevention of sport-related transmission | Krug A | Team sports | 1 | 2 | 0 | 0 | 0 | -1 | 2 | Low | (Strategy) Total number of people who requested testing was unknown. |
| COVID-19 outbreak among physicians at a Canadian curling bonspiel: a descriptive observational study | Burak KW | Team sports | 0 | 1 | 2 | 0 | 2 | -1 | 4 | Medium | - |
| Case Report: Assessing COVID-19 Transmission in Professional Volleyball in Germany, September to December 2020: An Epidemiological Study | Morath O | Team sports | 1 | 2 | 2 | 1 | 2 | -1 | 7 | High | (Timeliness) the start date is not clearly stated, but it appears to have gone at least to Day 9. |
| SARS-CoV-2 transmission during an indoor professional sporting event | Pauser J | Team sports | 1 | 2 | 0 | 0 | 2 | -1 | 4 | Medium | (Strategy) 88% participated |
| Mitigating a COVID-19 Outbreak Among Major League Baseball Players - United States, 2020 | Murray MT | Team sports | 1 | 2 | 2 | 2 | 2 | 0 | 9 | High | - |
